# Attentional biases toward threat: the concomitant presence of difficulty of disengagement and attentional avoidance in low trait anxious individuals

**DOI:** 10.3389/fpsyg.2014.00685

**Published:** 2014-07-01

**Authors:** Laura Sagliano, Luigi Trojano, Katja Amoriello, Michela Migliozzi, Francesca D’Olimpio

**Affiliations:** Department of Psychology, Second University of NaplesCaserta, Italy

**Keywords:** attentional bias, threat, anxiety, spatial attention, avoidance, disengagement

## Abstract

Attentional biases toward threats (ABTs) have been described in high anxious individuals and in clinical samples whereas they have been rarely reported in non-clinical samples (Bar-Haim et al., 2007; Cisler and Koster, 2010). Three kinds of ABTs have been identified (facilitation, difficulty of disengagement, and avoidance) but their mechanisms and time courses are still unclear. This study aimed to understand ABTs mechanisms and timing in low trait anxiety (LTA) and high trait anxiety (HTA) anxious individuals. In particular, in an exogenous cueing task we used threatening or neutral stimuli as peripheral cues with three presentation times (100, 200, or 500 ms). The main results showed that HTA individuals have an attentional facilitation bias at 100 ms (likely automatic in nature) whereas LTA individuals show attentional avoidance and difficulty to disengage from threatening stimuli at 200 ms (likely related to a strategic processing). Such findings demonstrate that threat biases attention with specific mechanisms and time courses, and that anxiety levels modulate attention allocation.

## INTRODUCTION

Different kinds of attentional biases toward threats (ABTs) have been described in high anxious individuals and in clinical samples, whereas they have been rarely reported in low anxiety individuals (Bar-Haim et al., 2007; Cisler and Koster, 2010). Indeed, when faced with fearful stimuli, high anxious individuals tend to detect them quickly (facilitation bias), at 100 and 200 ms of stimulus presentation times (PTs; Koster et al., 2006, 2007; Massar et al., 2011), and to remain anchored upon them (difficulty of disengagement), between 100 and 500 ms (Fox et al., 2001; Koster et al., 2004b, 2006; Massar et al., 2011), whereas non-anxious individuals seem to avoid the same stimuli (avoidance bias), after ∼200 ms (Koster et al., 2006, 2007). The disengagement bias toward threat has been observed in both high state anxiety individuals at rapid PTs (Fox et al., 2001) and high trait anxiety individuals at a slower PT (Fox, 2002). Moreover, Koster et al. (2006) showed that, after an early facilitation bias, high anxious individuals show a subsequent tendency to shift their attention away from threatening stimuli (avoidance bias). Therefore, the facilitation and the disengagement biases have been found in high trait anxiety individuals only, whereas the avoidance bias has been reported in both high and low trait anxious individuals.

Mathews (1990) proposed that the ABTs play an important role in maintaining high anxiety levels, as anxious individuals would be more likely to detect potential threats in the environment, which would increase their anxiety levels. However, different, and often contrasting, hypotheses have been subsequently put forward to explain ABTs (for a review, see Cisler and Koster, 2010). For instance, Williams et al. (1997) suggested that high and low anxious individuals differ in their attention allocation mechanisms in presence of threatening stimuli: high anxiety individuals direct attention toward threat while low anxiety individuals direct attention away from threat. Similarly, Eysenck et al. (2007) proposed that high anxious individuals show an impairment in attentional control, enhancing vigilance for threatening stimuli and inducing difficulties in disengaging attention from threat. According to a different point of view, the so-called vigilance–avoidance hypothesis of ABTs (Mogg et al., 2004), high anxious individuals tend to overestimate the stimuli’s threat value, and show an enhancement of automatic mechanisms detecting potential threats, but also tend to avoid further processing of stimuli closely matching their own phobic concerns.

More recently, Cisler and Koster (2010) proposed that, in high anxious individuals and in clinical samples, the three biases (facilitation, difficulty in disengagement, and avoidance) differ as regards: (i) type of processing (automatic or strategic), (ii) cognitive mechanisms (attentional control and emotion regulation goals), and (iii) neural bases (amygdala and prefrontal circuits). In detail, Cisler and Koster (2010) suggested that the automatic processing of attention, mediated by the amygdala, is responsible for detecting threatening stimuli and rapidly orienting attention toward them (facilitation bias). Strategic or conscious elaboration (mediated by the frontal cortex network) would be instead responsible for biased attention distribution (favoring allocation of attention on neutral stimuli: avoidance), and strategic attentional control (determining difficulties in disengagement). The model nicely explains findings (Koster et al., 2006) showing that high anxious (but not low anxious) individuals show different ABTs in response to aversive stimuli as a function of PTs: in particular, facilitation bias and difficulty in disengagement with short PTs, and an avoidance bias with longer PTs. The time course of ABTs has been confirmed by Massar et al. (2011), who found an early attentional engagement for threatening stimuli in high anxious individuals; however, the authors also found a slower disengagement from threat cues in all participants, irrespective of their trait anxiety levels, in contrast with ((s))Koster et al. (2006) observations about the lack of ABTs in low anxious individuals. Therefore, ((s))Massar et al. (2011) findings would be compatible with the idea that the ((s))Cisler and Koster (2010) model would also apply to non-anxious people, at least for threatening stimuli.

The idea that the same model can be applied to people with low and high anxiety is not consistent with recent data showing a differential modulation of hypervigilance (facilitation bias) and avoidance in high and low trait anxious individuals. Actually, using a conditioning procedure, Onnis et al. (2011) reported that high anxious participants showed an attentional facilitation when stimuli were presented for 200 ms and an attentional avoidance when stimuli were presented for 500 ms, whereas low anxious individuals showed an opposite attentional pattern, with an early tendency to divert attention from aversive stimuli (200 ms presentation) and a later orientation toward them (500 ms presentation). These data would confirm that facilitation and avoidance are characterized by distinct attentional mechanisms operating at different stages of information processing, but also would suggest that activation of such mechanisms is dependent on anxiety levels.

On the basis of the studies reviewed above, two questions are still open. First, ((s))Cisler and Koster (2010) hypothesis, according to which the facilitation bias is related to an early automatic processing whereas avoidance and disengagement biases are driven by later strategic elaboration, has not been directly tested in a study tapping all the three ABTs in a comprehensive within-subject paradigm. Second, it is not clear whether the same cognitive mechanisms can account for ABTs in low and high trait anxious individuals.

The present study aimed to tackle these issues by an experimental paradigm combining within- and between-subjects observation, in which threatening or neutral stimuli modulated explicit allocation of spatial attention. By using three PTs we could systematically explore: (i) whether facilitation, difficulty of disengagement and avoidance are specifically related to early or late time windows, as foreseen by ((s))Cisler and Koster (2010) model, within the same subjects, and (ii) whether the same pattern of ABTs can be observed in two groups of individuals with low or high trait anxiety, consistent with possible generalization of the model, independently from anxiety levels.

According to the original formulation of ((s))Cisler and Koster (2010) model we could expect to find a facilitation bias at the shortest PTs, and difficulty of disengagement and avoidance bias at the longest PT in high anxious individuals, and no bias in low anxious participants. However, the present study would also make possible to find the same ABTs, with the same time course, in low anxious individuals too, thus suggesting that ((s))Cisler and Koster (2010) model can apply irrespective of anxiety level, and can be considered as a general model of emotion-related modulation of attentional resources, reflecting adaptive (or maladaptive) response mechanisms. It is also possible to find partial discrepancies between high and low anxious individuals, compatible with the idea that high anxiety levels can affect deployment of attentional resources over environmental features, whereas the low anxious pattern of ABTs might reflect the most advantageous response modality to possible threats.

## MATERIALS AND METHODS

### PARTICIPANTS

Participants were 95 non-clinical female undergraduate students from the Second University of Naples, dwelling in South Italy (age range = 20–33 years, mean age = 23.85, SE = 3.2). As in previous studies on ABTs (e.g., Leyman et al., 2009; De Raedt et al., 2010), only female participants were included in the study to ensure maximum homogeneity of the sample, and because women are considered to show greater facility in decoding non-verbal messages and to rate their emotions more intensely than males (Killgore and Yurgelun-Todd, 2001).

The participants were assigned to one of two groups according to their anxiety scores on the Trait subscale of State-Trait Anxiety Inventory (STAI; Spielberger et al., 1983): following previous studies (Onnis et al., 2011), participants with STAI-Trait score < 35 were included in the LTA group, and participants with a STAI-T score > 49 were included in the HTA group; individuals with intermediate scores (35–49) were excluded from the study. All subjects were right-handed, had normal or corrected-to-normal vision, and were naive to purposes and predictions of the experiment. Participants gave their written informed consent to take part in the experiment on a voluntary basis, without receiving any reward.

### PROCEDURE

Participants completed state and trait versions of the STAI-Y and, then, were asked to perform a modified version of the Posner Task.

### MATERIALS

#### State-Trait Anxiety Scale

The STAI-Y (Spielberger et al., 1983) consists of two 20-item scales aiming at measuring state and trait anxiety. The STAI-State subscale requires respondents to rate how they feel “right now… at this moment” using a 4-point scale (1 = not at all, 4 = very much so) in response to a series of self-descriptive statements. The STAI-Trait subscale, used here to allocate subjects to LTA or HTA groups, asks respondents to rate how they “generally” feel using a 4-point scale (1 = almost never, 4 = almost always) in response to a series of self-descriptive statements. These subscales have been demonstrated to be valid and to have solid psychometric properties (Spielberger et al., 1983).

#### Exogenous cueing task

Participants were presented with a dot detection task driven by an exogenous (threatening or neutral) spatial cue; this paradigm is a modified version of the Posner (1980). Each trial began with a fixation cross (+) flanked by two blank squares (340 × 340 pixel) on its right and left side. After 750 ms, a cue (a threatening or non-threatening image; 300 × 300 pixel) appeared in one of the two square for 100, 200, or 500 ms in randomized order, followed by a dot (1 cm) presented in one of the two squares, in the same (valid trial) or in the opposite (invalid trial) position as the cue.

Images used as cues were selected from a larger sample 150 images consisting of familiar scenes of animals, people or natural events in order to maximize ecological validity. In a preliminary phase, images were shown, one at a time, on a pc monitor to 30 undergraduate students (age range: 20–30), who were asked to judge threat degree of each stimulus on a scale from 0 (not threatening) to 4 (very threatening) by pressing a corresponding key on the pc keyboard. For the present experiment we used the 20 images judged as most threatening (mean score of threat degree = 2.9; range = 2.5–4), and the 20 images judged as least threatening (mean score of threat degree = 0.7; range = 0–1). Each stimulus appeared at least once in right and left squares.

**FIGURE 1:**
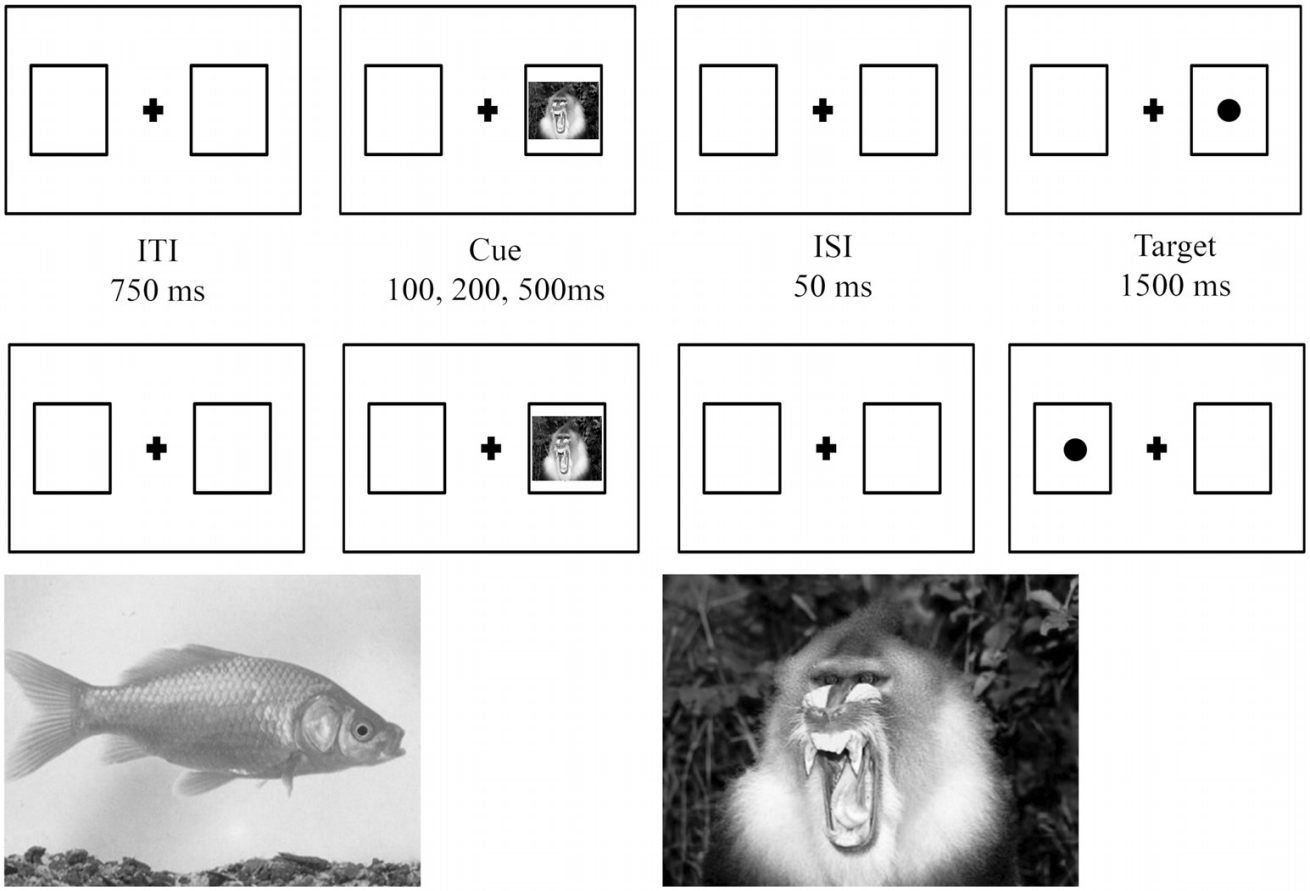
**Schematic overview of valid (first row) and invalid trials (middle row).** Examples of non-treatening (left) or threatening (right) stimuli are depicted in the bottom row.

Valid (*n* = 192, 80%; 96 threatening and 96 non-threatening) and invalid (*n* = 48, 20%; 24 threatening and 24 non-threatening) trials were presented in a randomized order for a total of 240 trials (**Figure 1**).

Participants were required to respond, as fast and accurately as possible, pressing a right key (m) on the keyboard when the target (dot) appeared on the right and a left key (z) when the target appears on the left. Both accuracy and response times (RTs) were recorded.

### DATA ANALYSIS

A preliminary multivariate analysis of variance (MANOVA) with anxiety group (LTA, HTA) as the independent factor was conducted on age and anxiety levels, to characterize the two samples.

After removing outliers (RT<150 and >1000; Koster et al., 2004a), raw RTs for correct trials were analyzed by a mixed analysis of variance (ANOVA) 2X2X2X3 with one between-subject factor (anxiety group: LTA, HTA) and three within-subject factors (valence: threatening, non-threatening; validity: valid, invalid; PTs: 100, 200, 500 ms).

Planned contrasts with Bonferroni correction were used to compare RTs for threatening vs. non-threatening stimuli.

As suggested by Koster et al. (2006), for the analysis of single ABTs we calculated the facilitation score (RTvalid/non-threatening cue – RTvalid/threatening cue) and the disengagement score (RTinvalid/threatening cue – RTinvalid/non-threatening cue). A positive facilitation score indicates an early enhanced attentional capture by threatening cues compared with non-threatening cues (facilitation bias). A positive value on disengagement score indicates stronger attentional holding by threatening cues compared with non-threatening ones (disengagement bias). Negative values of both scores indicate a tendency to avoid threatening stimuli (avoidance bias). A value not different from zero at either score means lack of ABTs (i.e., no difference in processing of threatening vs. non-threatening cues).

A MANOVA with anxiety group (LTA, HTA) as independent factor was conducted on bias scores. Univariate analyses and planned comparisons with Bonferroni correction were then executed.

Single-sample *t*-test comparisons were used to evidence whether bias scores were significantly different from zero.

## RESULTS

### GROUP CHARACTERISTICS

On the basis of the results of the Trait subscale of the STAI, 27 participants were included in the LTA group and 28 in the HTA group, whereas 40 subjects were excluded from the study.

The MANOVA with trait anxiety group as the independent factor, and age, state and trait anxiety scores as outcome variables confirmed that the HTA group had significantly higher scores compared to the LTA group both in trait anxiety [HTA = 59.18; LTA = 31.30; *F*(1,53) = 325.92, *p* < 0.001, $\eta_{p}^{2}$ = 0.86] and in state anxiety [HTA = 46.68; LTA = 31.11; *F*(1,53) = 40.06, *p* < 0.001, $\eta_{p}^{2}$ = 0.43], whereas the two groups did not differ in age [HTA = 22.64; LTA = 23.26; *F*(1,53) = 0.69, *p* = 0.40, $\eta_{p}^{2}$ = 0.01].

### DOT DETECTION TASK

Means and standard deviations for correct RTs are reported in **Table 1**.

**Table 1:** Mean and SE of the RTs in the Modified Posner Task as a function of anxiety group, validity, valence, and PT.

			LTA		HTA	

PT	Validity	Valence	Mean	SE	Mean	SE
100	Invalid	Threatening	402.59	13.37	394.44	13.13
		Non-threatening	401.63	12.75	380.79	12.52
						
	Valid	Threatening	354.45	11.53	334.52	11.32
		Non-threatening	354.69	11.62	340.12	11.41
						
200	Invalid	Threatening	395.60	12.58	384.54	12.35
		Non-threatening	378.81	13.50	378.78	13.26
						
	Valid	Threatening	332.88	11.76	309.64	11.54
		Non-threatening	324.04	11.07	311.52	10.87
						
500	Invalid	Threatening	373.15	12.67	357.73	12.44
		Non-threatening	373.17	12.64	364.94	12.41
						
	Valid	Threatening	313.83	10.87	299.00	10.67
		Non-threatening	309.84	9.97	293.04	9.79


The ANOVA on RTs showed that all within-subject main effects were significant. In particular, the effect of Validity [*F*(1,53) = 293.98, *p* < 0.001, $\eta_{p}^{2}$ = 0.85] was related to faster responses for valid (*M* = 323.13) than for invalid trials (*M* = 382.18; *p* < 0.001); the effect of Valence [*F*(1,53) = 5.13, *p* = 0.03, $\eta_{p}^{2}$ = 0.09] was due to faster responses for non-threatening (*M* = 350.95) than for threatening stimuli (*M* = 354.36; *p* = 0.03); last, the effect of PT [*F*(2,106) = 83.42, *p* < 0.001, $\eta_{p}^{2}$ = 0.61] was related to faster responses for longer PTs (100 ms = 370.40; 200 ms = 351.97; 500 ms = 335.59; all different from each other at *p* < 0.001). The main effect of Group was not significant [*F*(1,53) = 0.78, *p* = 0.38, $\eta_{p}^{2}$ = 0.01].

We also observed two significant interactions: Validity × PT interaction [*F*(2,106) = 8.91, *p* < 0.01, $\eta_{p}^{2}$ = 0.14], and Valence × PT × Validity interaction [*F*(2,106) = 3.85, *p* = 0.02, $\eta_{p}^{2}$ = 0.07], whereas all other interactions were not significant. Planned comparison on the Valence × PT × Validity interaction revealed significant shorter RTs for non-threatening (*M* = 378.80) compared to threatening stimuli (*M* = 390.06) only for invalid trials at 200 ms. No other significant difference emerged.

The MANOVA on bias scores showed a significant effect of Group factor on attentional facilitation at 200 ms [*F*(1,53) = 4.75, *p* = 0.03, $\eta_{p}^{2}$ = 0.08], as HTA individuals showed a positive facilitation bias (*M* = 1.88; SE = 3.45) and LTA showed a negative facilitation bias (*M* = -8.84; SE = 3.51).

**FIGURE 2:**
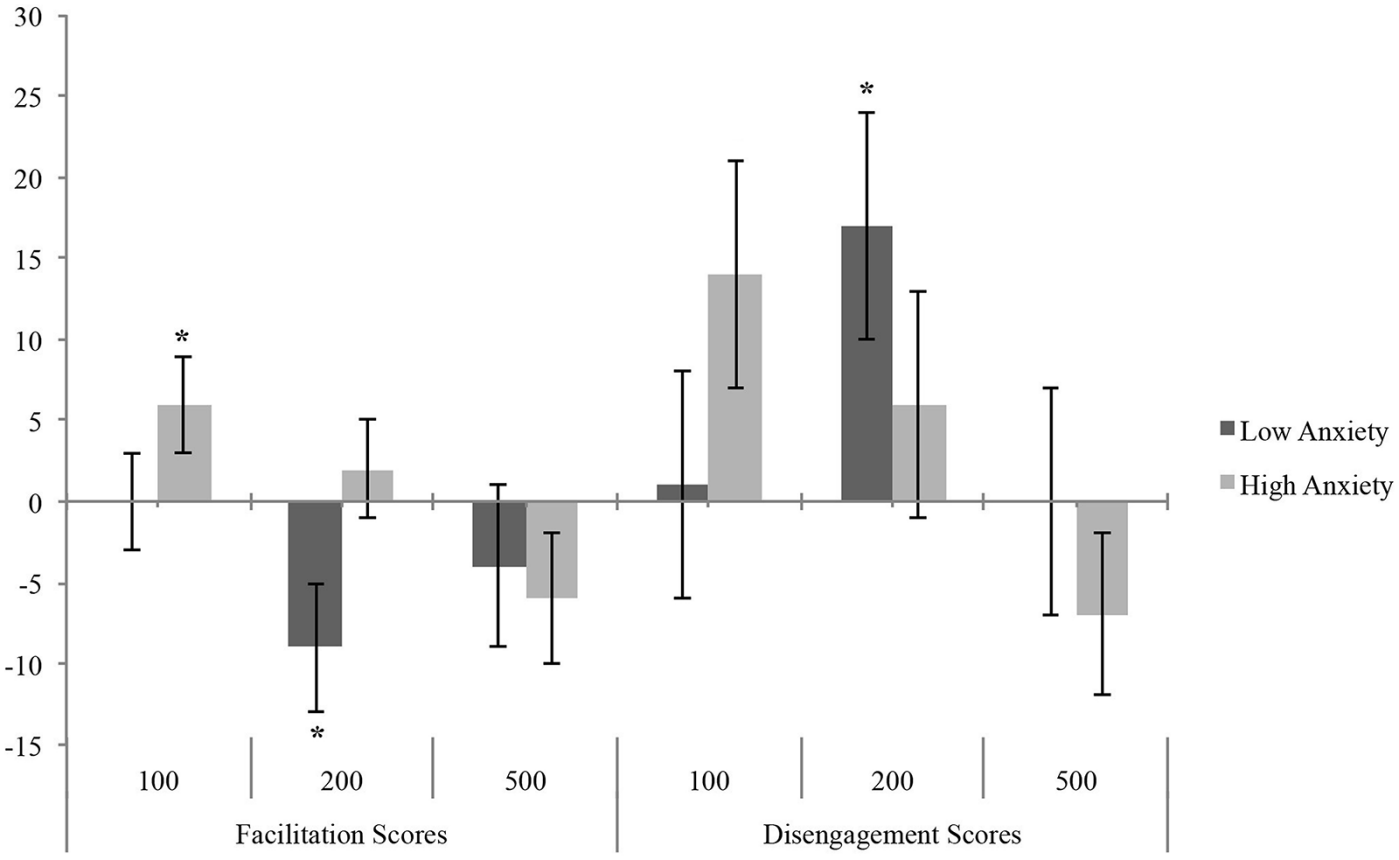
**Mean (and standard error) of Bias Score as a function of anxiety in LTA and HTA groups.** Asterisks indicate significant differences between ABT and zero (*p < 0.05).

Furthermore, one-sample *t*-tests on bias scores, in comparison to zero (Koster et al., 2006), revealed that LTA (**Figure 2**) showed attentional disengagement bias [*t*(26) = 2.38, *p* = 0.02] and avoidance [*t*(26) = -2.27, *p* = 0.03] at 200 ms; instead, HTA showed a significant facilitation bias at 100 ms [*t*(27) = 2.06, *p* = 0.049], whereas the difficulty in disengagement at 100 ms fell short of the significance level [*t*(27) = 1.97, *p* = 0.059].

## DISCUSSION

Our study investigating ABTs in both low and high trait anxiety individuals, identified a significant facilitation bias at the shortest PT (100 ms) in HTA individuals, in line with previous studies (Koster et al., 2006, 2007). This bias is compatible with the idea that an automatic processing system is responsible for detecting and orienting attention toward threat (Mathews and Mackintosh, 1998; Mogg and Bradley, 1998; Cisler and Koster, 2010).

The lack of attentional bias in HTA individuals at longer PTs (200 and 500 ms) is not consistent with findings reported by Koster et al. (2006) in HTA, and by Koster et al. (2007) in normal individuals with intermediate levels of trait anxiety. It should be remembered that ABTs at 200 ms PT have also been reported in spider-fearful individuals with high fear (Mogg and Bradley, 2006), when presented with fear congruent stimuli. It is important to underline that the discrepancies between the present results and those reported by Koster et al. (2006, 2007) and by Mogg and Bradley (2006) might be ascribed to the different characteristics of the stimuli employed in the experimental paradigms. In their studies Koster et al. (2006, 2007) used the International Affective Picture System (IAPS; Lang et al., 2005) scenes and intensive, high threat stimuli, as the authors themselves underlined (for instance, “mutilated face” with strong negative valence and high arousal value), whereas Mogg and Bradley (2006) used stimuli (spiders) with strong negative valence and high arousal value for the specific sample they assessed. Here, we employed familiar stimuli (such as animals, everyday scenes, and common objects), without selecting high intensity threatening items as in previous studies. It is therefore entirely plausible that the stimuli used in the present study were less liable to produce avoidance and disengagement biases in high anxious individuals. In fact, according to the dual competition framework (Pessoa, 2009), threat-related stimuli carry affective significance, which alters performance by strengthening sensory representations at the perceptual level and by prioritizing attention at the executive level. Although threat consistently leads to prioritize perceptual processing, its effect on executive control dramatically depends on the level of threat: high threat stimuli would enhance processing of the threat (hard prioritization), while low threat stimuli would determine a slight improvement of threat processing (soft prioritization). In line with this framework (Pessoa, 2009), the threat intensity of the stimuli used in the present study might have interacted with anxiety levels, determining a different prioritization in the HTA and LTA groups.

The main finding of the present study was indeed the divergence between the pattern of ABTs found in HTA and in LTA groups. A difference between high and low anxious individuals in attentional allocation mechanisms has been already hypothesized by Williams et al. (1997), who suggested that high anxiety would be characterized by a facilitation bias, whereas low anxiety individuals would be particularly characterized by avoidance bias. As recalled above, our experimental paradigm allowed us to detect only a significant facilitation bias in HTA individuals, whereas we found both difficulty in disengagement and attentional avoidance in the LTA group. Several previous studies on low anxiety individuals did report threat-related attentional biases (Mogg et al., 1994; Yiend and Mathews, 2001; Massar et al., 2011), whereas other studies only detected attentional avoidance (MacLeod and Mathews, 1988) or difficulty in disengagement (Massar et al., 2011). To the best of our knowledge, there is no previous evidence about co-occurrence of both biases in LTA at a specific time window (200 ms PT), but not at very rapid (100 ms) or longer (500 ms) PTs.

The specific time course of difficulty in disengagement and attentional avoidance, observed in LTA only, would exclude that these findings can be ascribed to a general slowing of responding to subsequent target stimuli caused by threat cues in exogenous cueing task (Mogg et al., 2008). According to an alternative interpretation, the simultaneous presence of difficulty of disengagement and avoidance at 200 ms in LTA would only reflect a form of cognitive freezing, as suggested by Fox et al. (2001) or non-attentional behavioral freezing, as suggested by Clarke et al. (2013). Freezing is an early response to detected danger throughout the animal kingdom that increase the chances of survival in threatening situations (LeDoux, 1996), but it has freezing-like responses have been also detected in normal human individuals engaged in concurrent cognitive tasks (Sagliano et al., 2014). It can be argued that the delayed responses to threatening stimuli in both valid and invalid trial could reflect a cognitive form of the freezing response, but it would remain to explain the reason why only LTA showed these biases, and only at 200 ms.

The finding of a specific disengagement bias at 200 ms is congruent with ((s))Cisler and Koster (2010) model, positing that attentional facilitation is driven by automatic processing, while the disengagement bias and the attentional avoidance reflect strategic orienting of attention. In this perspective, the presence of such biases in LTA would support the idea that ((s))Cisler and Koster (2010) model is not specific for the clinical sample but it can be applied to all individuals, independently from their anxiety level. However, as suggested by Koster et al. (2006), HTA individuals are characterized by an oversensitive threat appraisal system that leads to overestimate valence of threatening stimuli. This causes a shift of attention toward moderately threatening stimuli (facilitation bias) in these individuals, whereas LTA individuals do not show the same enhanced, rapid detection of threats, and are able to strategically avoid threatening stimuli and yet to take such stimuli under attentional control.

Disengagement bias may serve to maintain and enhance anxiety states (Fox et al., 2001). In contrast, avoidance bias, i.e., the ability to rapidly disengage from threat-related material once identified, may be a useful mechanism to keep anxiety levels under control.

Several studies (Beck and Clark, 1997) suggested that a top-down modification of attention allocation would reduce the risk of negative consequences from threat, thus resulting in an attentional avoidance of threatening stimuli. However, it should be underlined that LTA individuals showed at the same time window (200 ms) both attentional avoidance and difficulty to disengagement, an apparently paradoxical finding (see Cisler and Koster, 2010). Nevertheless, on the basis of the distinction between overt and covert attentional mechanisms (Posner, 1980). Weierich et al. (2008) argued that individuals might overtly avoid the threat and covertly maintain their attention on it. This might represent the most effective method to deal with potential threats, without activating strong emotion-related cognitive and neural processes. This ability to react to threat might reduce individual vulnerability to adverse events. Recently, Min et al. (2012) suggested that the ability to respond to stress and adversity, together with LTA levels, might reduce the risk to develop psychiatric disorder; Min et al. (2012) also suggested that evaluation and management of trait anxiety can enhance patient’s resilience and improve treatment of depression and anxiety disorders. Our results are substantially in line with these statements. Indeed, the difference between high and low anxious individuals revealed in this study is compatible with the idea that the ability to simultaneously control and avoid threat showed by LTA might be considered the most advantageous response modality, likely allowing to minimize negative emotional responses to threats and, possibly, the risk of developing clinically relevant anxiety.

In other words, this specific pattern of ABTs might reflect the differences between HTA and LTA’s behavior, and help comprehending why some individuals are characterized by low levels of anxiety.

The lack of analogous findings in LTA in previous studies might be ascribed to the specific methodological procedures adopted here, as regards the stimuli (we used familiar items to assess responses to plausible threats), the experimental paradigm (we used three randomized PTs to avoid participants prepare their responses), and the sample selection (we selected a gender-homogeneous sample, thus minimizing variability, on the basis of well defined cut-off values for low or high trait anxiety). These methodological choices likely contributed to put in evidence previously unreported findings in LTA, but also impose some caveats in generalizing the present results. Future studies will have to verify whether the same pattern is present in male individuals, and, above all, to take into account the possible effects of stimuli’s valence also assessing physiological correlates of threat processing. Moreover, future studies might also take into account the possible interaction of depressive mood with ABTs, although available evidence would suggest that depressed individuals usually show ABTs at PT longer than those used in the present study (Mogg and Bradley, 2005).

## Conflict of Interest Statement

The authors declare that the research was conducted in the absence of any commercial or financial relationships that could be construed as a potential conflict of interest.
